# Matrices of Spin-Orbit Interaction in the Electron Configurations *np*^2^ *n*′ *p* and *np*^4^ *n*′ *p*

**DOI:** 10.6028/jres.067A.052

**Published:** 1963-12-01

**Authors:** Jack L. Tech

## Abstract

The matrices of spin-orbit interaction in the *p*^2^ *p* and *p*^4^ *p* electron configurations have been calculated. The matrices have been checked by showing that their eigenvalues, calculated by use of an IBM 7090, agree with the correct eigenvalues known from the theory of *jj*-coupling. For the sake of completeness, the matrices of electrostatic interaction for these configurations are also given.

## 1. Introduction

In connection with our analysis of the first spectrum of bromine, reported in the preceding paper, it became desirable to know the energy matrices in intermediate coupling for the configuration *np*^4^ *n*′*p*. Since the matrices of spin-orbit interaction for this configuration have not appeared in the literature it was decided, at the suggestion of Professor Racah, to calculate them. The primary use of such interaction matrices is to obtain approximate predicted values for the energy levels of atomic systems. The eigenvalues and eigenvectors of the matrices represent, respectively, the energy levels and wave functions of the atomic system.

The predicted levels are a great aid in the problem of locating and identifying the observed energy levels. Until recently, most comparisons with observations were carried out in the approximation of pure Russell-Saunders coupling, employing in the theoretical calculations only the matrices of the electrostatic interaction. But the availability of electronic digital computers now renders it practical to include the effects of spin-orbit interaction in the calculations.

## 2. Spin-Orbit Interaction

The matrices of spin-orbit interaction for the configuration *np*^2^ *n′ p* are given in table 3. Since the energy matrix is diagonal in *J*, the nondiagonal elements occur only between levels with the same *J* value. There is thus one matrix for each possible value of *J*. The rows and columns of the matrices are specified by the name of the term in *LS*-coupling notation, the terms in parentheses denoting the parent terms in *np*^2^. The elements of these matrices are linear combinations of the spin-orbit integrals *ζ* and *ζ*′, where *ζ* is written for *ζ_np_* and *ζ*′ for *ζ_n_*′*_p_*. Both these integrals are positive. Their coefficients were calculated following the methods outlined by Fano and Racah [1].^1^ Values of the $\bar{W}$-coefficients, or 6-*j* symbols, needed for these calculations were obtained from the compilation of Rotenberg et al. [2]. The matrices have been checked by using an IBM 7090 to determine their eigenvalues and showing them to agree with the correct eigenvalues known from the theory of *jj*-coupling [cf. 3, ch. 10].

The matrices for the *np*^4^ *n*′ *p* configuration are identical to the above except that in this case the sign of the spin-orbit coupling parameter *ζ_np_* is reversed [3, p. 299].

## 3. Electrostatic Interaction

To obtain the complete energy matrices in intermediate coupling, the electrostatic matrices must be added to the spin-orbit matrices of table 3. The matrices of electrostatic interaction in Russell-Saunders coupling are well-known for the *np*^2^ *n*′ *p* and *np*^4^ *n*′ *p* configurations, and we give them here in tables 1 and 2 for the sake of completeness. The elements of the matrices are expressed as linear combinations of the usual parameters *F_k_* and *G_k_*, which are defined as certain integrals over radial wave functions [3, p. 177]. We calculated the coefficients of these parameters from the general tables given by Slater [4, vol. II, appendix 21]. Terms of a kind that occur only once in these configurations have no matrix elements connecting them to other terms; the diagonal components give the energies directly. Since there are two ^2^D° terms, however, there are matrix components between them, and the electrostatic energies of the two terms are found as the eigenvalues of the corresponding matrix. The same holds also for the three ^2^P° terms.

Following Slater, the energies are stated in terms of the average energy, *E*_av_≡*E*, which represents the center of gravity of the terms of the configuration, each term being assigned the weight (2*S*+1) (2L + 1). The energy expressions can easily be converted, if desired, so as to conform to the usage of Condon and Shortley. In this case, *E* is replaced in the energy expressions by the following quantities:

$$\begin{array}{l} \text{Configuration}\;E\\ \begin{array}{l} np^{2}n^{\prime }p\;(F_{0}+2F_{0}^{{\;}^{\prime }})-2F_{2}-\frac{1}{3}G_{{\;}^{0}}^{{\;}^{\prime }}-\frac{10}{3}G_{{\;}^{2}}^{{\;}^{\prime }}\\ np^{4}n^{\prime }p\;(6F_{0}+4F_{0}^{{\;}^{\prime }})-12F_{2}-\frac{2}{3}G_{{\;}^{0}}^{{\;}^{\prime }}-\frac{20}{3}G_{{\;}^{2}}^{{\;}^{\prime }} \end{array} \end{array}$$

Here, as in tables 1 and 2, the parameters without primes refer to the (*np*, *np*) interactions while those with primes refer to the (*np, n*′*p*) interactions. In accordance with TAS, the subscripted parameters are defined in terms of the corresponding superscripted parameters as follows:

$$\begin{array}{l} F_{2}(p,p)=\frac{1}{25}F^{2}(p,p)\\ F_{2}(p,p^{\prime })=\frac{1}{25}F^{2}(p,p^{\prime })\\ G_{0}(p,p^{\prime })=G^{0}(p,p^{\prime })\\ G_{2}(p,p^{\prime })=\frac{1}{25}G^{2}(p,p^{\prime }) \end{array}$$

## 4. Summary

The matrices of spin-orbit interaction for the configurations *np*^2^ *n*′ *p* and *np*^4^ *n*′ *p* have been calculated and are presented in table 3. The matrices of electrostatic interaction are also given, in tables 1 and 2. The complete energy matrices for these configurations can thus be formed in the approximation that neglects spin-spin and spin-other-orbit interactions as well as configuration mixing.

The configurations under discussion occur in a number of important neutral and ionized atoms, including the halogens and Bi, Sb, As, P, N, and their isoelectronic sequences. The sets of energy levels associated with these configurations are known to different stages of completeness for the various atoms. A systematic, comparative study of these atoms is being launched. A similar study is contemplated for the *np*^2^*n*′*d* and *np*^4^*n*′*d* configurations, whose spin-orbit matrices are presently being calculated and will be reported at a later time.

## Figures and Tables

**Table 1:** Electrostatic energies for np^2^n′p

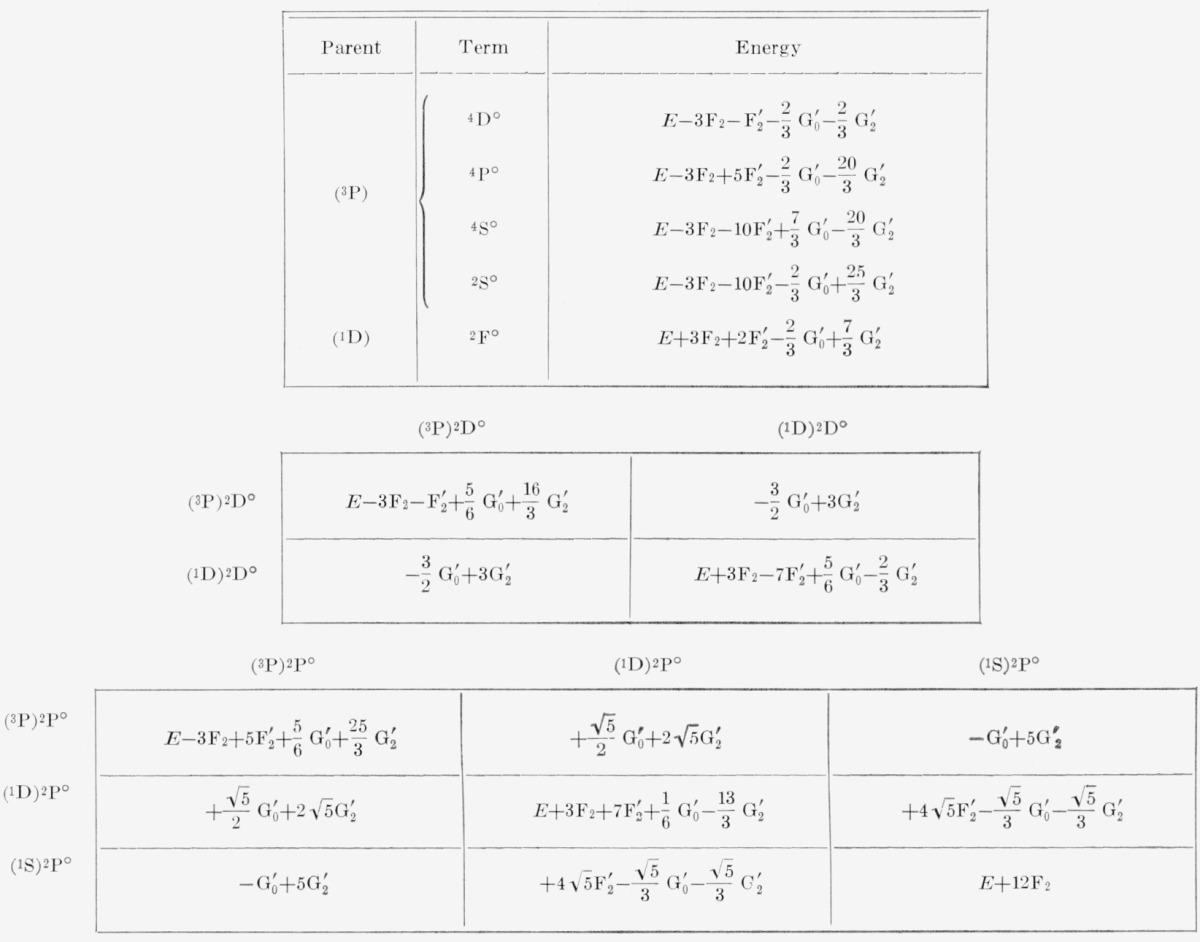

**Table 2:** Electrostatic energies for np^4^n′p

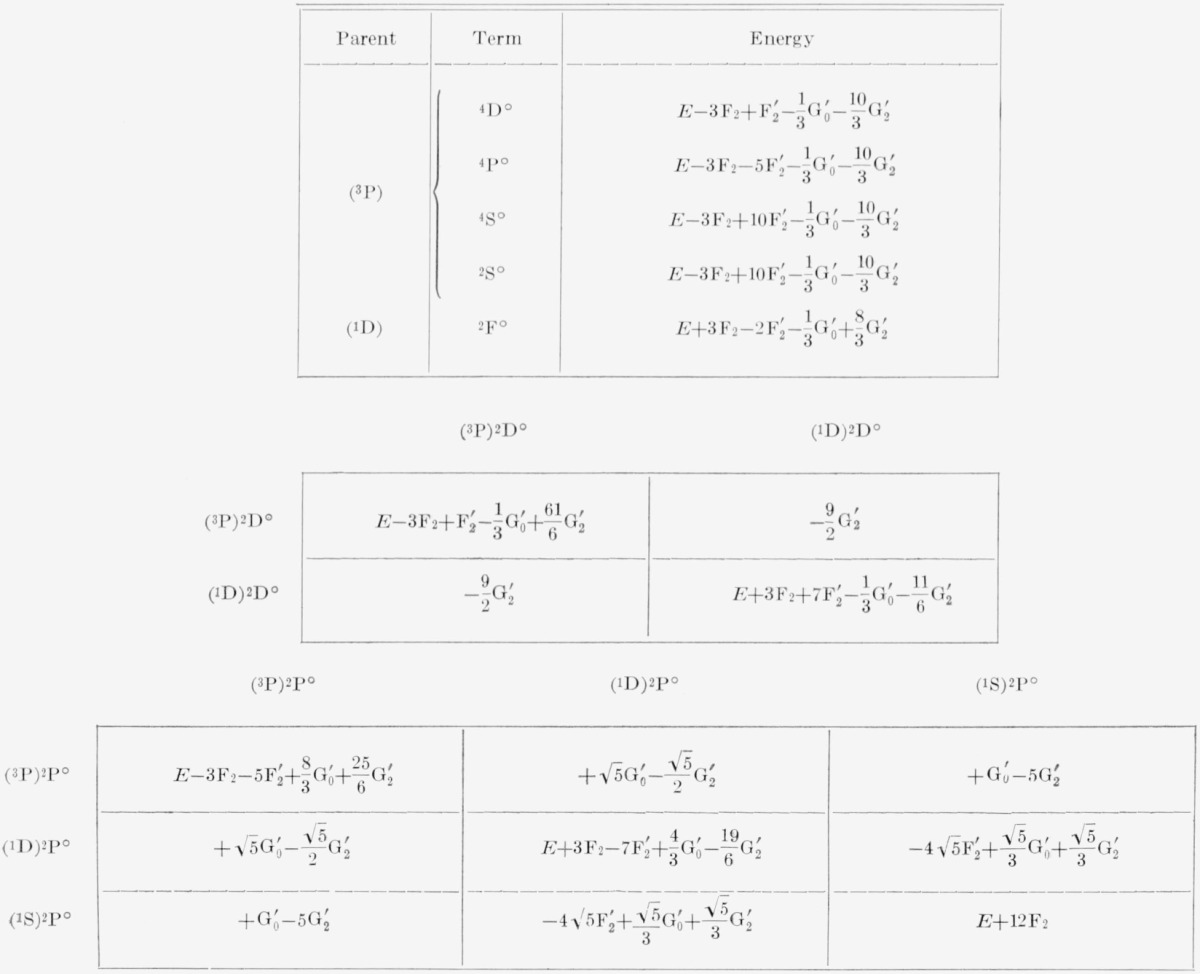

**Table 3:** Matrices of spin-orbit interaction for the configuration np*^2^*n′p

$J=\frac{7}{2}$	(^1^D)^2^F°	(^3^P)^4^D°
(^1^D)^2^F°	$+\frac{1}{2}\zeta^{\prime }$	$+\frac{\sqrt{2}}{2}\zeta$
(^3^P)^4^D°	$+\frac{\sqrt{2}}{2}\zeta$	$+\frac{1}{2}(\zeta +\zeta^{\prime })$

$J=\frac{5}{2}$	(^1^D)^2^F°	(^3^P)^4^D°	(^3^P)^2^D°	(^1^D)^2^D°	(^3^P)^4^P°
(^1^D)^2^F°	$-\frac{2}{3}\zeta^{\prime }$	$+\frac{1}{3}\zeta$	$-\frac{\sqrt{14}}{6}\zeta$	$-\frac{\sqrt{14}}{6}\zeta^{\prime }$	0
(^3^P)^4^D°	$+\frac{1}{3}\zeta$	$-\frac{1}{12}(\zeta +\zeta^{\prime })$	$-\frac{\sqrt{14}}{12}(\zeta -2\zeta^{\prime })$	$+\frac{\sqrt{14}}{12}\zeta$	$+\frac{\sqrt{21}}{12}(\zeta -\zeta^{\prime })$
(^3^P)^2^D°	$-\frac{\sqrt{14}}{6}\zeta$	$-\frac{\sqrt{14}}{12}(\zeta -2\zeta^{\prime })$	$+\frac{1}{6}(2\zeta -\zeta^{\prime })$	$+\frac{1}{6}\zeta$	$+\frac{\sqrt{6}}{12}(\zeta +2\zeta^{\prime })$
(^1^D)^2^D°	$-\frac{\sqrt{14}}{6}\zeta^{\prime }$	$+\frac{\sqrt{14}}{12}\zeta$	$+\frac{1}{6}\zeta$	$+\frac{1}{6}\zeta^{\prime }$	$+\frac{\sqrt{6}}{4}\zeta$
(^3^P)^4^P°	0	$+\frac{\sqrt{21}}{12}(\zeta -\zeta^{\prime })$	$+\frac{\sqrt{6}}{12}(\zeta +2\zeta^{\prime })$	$+\frac{\sqrt{6}}{4}\zeta$	$+\frac{1}{4}(\zeta -\zeta^{\prime })$

$J=\frac{3}{2}$	(^3^P)^4^D°	(^3^P)^2^D°	(^1^D)^2^D°	(^3^P)^4^P°	(^3^P)^2^P°	(^1^D)^2^P°	(^1^S)^2^P°	(^3^P)^4^S°
(^3^P)^4^D°	$-\frac{1}{2}(\zeta +\zeta^{\prime })$	$-\frac{1}{4}(\zeta -2\zeta^{\prime })$	$+\frac{1}{4}\zeta$	$+\frac{1}{3}(\zeta -\zeta^{\prime })$	$-\frac{\sqrt{5}}{12}(\zeta +2\zeta^{\prime })$	$+\frac{1}{12}\zeta$	$-\frac{\sqrt{5}}{3}\zeta$	0
(^3^P)^2^D°	$-\frac{1}{4}(\zeta -2\zeta^{\prime }$	$-\frac{1}{4}(2\zeta -\zeta^{\prime })$	$-\frac{1}{4}\zeta$	$+\frac{1}{12}(\zeta +2\zeta^{\prime })$	$+\frac{\sqrt{5}}{12}(2\zeta +\zeta^{\prime })$	$+\frac{1}{12}\zeta$	$-\frac{\sqrt{5}}{3}\zeta$	0
(^1^D)^2^D°	$+\frac{1}{4}\zeta$	$-\frac{1}{4}\zeta$	$-\frac{1}{4}\zeta^{\prime }$	$+\frac{1}{4}\zeta$	$-\frac{\sqrt{5}}{4}\zeta$	$-\frac{3}{4}\zeta^{\prime }$	0	0
(^3^P)^4^P°	$+\frac{1}{3}(\zeta -\zeta^{\prime })$	$+\frac{1}{12}(\zeta +2\zeta^{\prime })$	$+\frac{1}{4}\zeta$	$-\frac{1}{6}(\zeta +\zeta^{\prime })$	$-\frac{\sqrt{5}}{12}(\zeta +2\zeta^{\prime })$	$+\frac{5}{12}\zeta$	$+\frac{\sqrt{5}}{3}\zeta$	$+\frac{\sqrt{10}}{6}(\zeta -\zeta^{\prime })$
(^3^P)^2^P°	$-\frac{\sqrt{5}}{12}(\zeta +2\zeta^{\prime })$	$+\frac{\sqrt{5}}{12}(2\zeta +\zeta^{\prime })$	$-\frac{\sqrt{5}}{4}\zeta$	$-\frac{\sqrt{5}}{12}(\zeta -2\zeta^{\prime })$	$+\frac{1}{12}(2\zeta +\zeta^{\prime })$	$+\frac{\sqrt{5}}{12}\zeta$	$+\frac{1}{3}\zeta$	$+\frac{\sqrt{2}}{6}(\zeta +2\zeta^{\prime })$
(^1^D)^2^P°	$+\frac{1}{12}\zeta$	$+\frac{1}{12}\zeta$	$-\frac{3}{4}\zeta^{\prime }$	$+\frac{5}{12}\zeta$	$+\frac{\sqrt{5}}{12}\zeta$	$-\frac{1}{4}\zeta^{\prime }$	0	$+\frac{\sqrt{10}}{6}\zeta$
(^1^S)^2^P°	$-\frac{\sqrt{5}}{3}\zeta$	$-\frac{\sqrt{5}}{3}\zeta$	0	$+\frac{\sqrt{5}}{3}\zeta$	$+\frac{1}{3}\zeta$	0	$+\frac{1}{2}\zeta^{\prime }$	$-\frac{\sqrt{2}}{3}\zeta$
(^3^P)^4^S°	0	0	0	$+\frac{\sqrt{10}}{6}(\zeta -\zeta^{\prime })$	$+\frac{\sqrt{2}}{6}(\zeta +2\zeta^{\prime })$	$+\frac{\sqrt{10}}{6}\zeta$	$-\frac{\sqrt{2}}{3}\zeta$	0

$J=\frac{1}{2}$	(^3^P)^4^D°	(^3^P)^4^P°	(^3^P)^2^P°	(^1^D)^2^P°	(^1^S)^2^P°	(^3^P)^2^S°
(^3^P)^4^D°	$-\frac{3}{4}(\zeta +\zeta^{\prime })$	$+\frac{\sqrt{5}}{12}(\zeta -\zeta^{\prime })$	$-\frac{\sqrt{10}}{12}(\zeta +2\zeta^{\prime })$	$+\frac{\sqrt{2}}{12}\zeta$	$-\frac{\sqrt{10}}{3}\zeta$	0
(^3^P)^4^P°	$+\frac{\sqrt{5}}{12}(\zeta -\zeta^{\prime })$	$-\frac{5}{12}(\zeta +\zeta^{\prime })$	$-\frac{\sqrt{2}}{12}(\zeta -2\zeta^{\prime })$	$+\frac{\sqrt{10}}{12}\zeta$	$+\frac{\sqrt{2}}{3}\zeta$	$-\frac{1}{3}(\zeta +2\zeta^{\prime })$
(^3^P)^2^P°	$-\frac{\sqrt{10}}{12}(\zeta +2\zeta^{\prime })$	$-\frac{\sqrt{2}}{12}(\zeta -2\zeta^{\prime })$	$-\frac{1}{6}(2\zeta -\zeta^{\prime })$	$-\frac{\sqrt{5}}{6}\zeta$	$-\frac{2}{3}\zeta$	$+\frac{\sqrt{2}}{6}(2\zeta +\zeta^{\prime })$
(^1^D)^2^P°	$+\frac{\sqrt{2}}{12}\zeta$	$+\frac{\sqrt{10}}{12}\zeta$	$-\frac{\sqrt{5}}{6}\zeta$	$+\frac{1}{2}\zeta^{\prime }$	0	$-\frac{\sqrt{10}}{6}\zeta$
(^1^S)^2^P°	$-\frac{\sqrt{10}}{3}\zeta$	$+\frac{\sqrt{2}}{3}\zeta$	$-\frac{2}{3}\zeta$	0	$-\zeta^{\prime }$	$+\frac{\sqrt{2}}{3}\zeta$
(^3^P)^2^S°	0	$-\frac{1}{3}(\zeta +2\zeta^{\prime })$	$+\frac{\sqrt{2}}{6}(2\zeta +\zeta^{\prime })$	$-\frac{\sqrt{10}}{6}\zeta$	$+\frac{\sqrt{2}}{3}\zeta$	0

## Footnotes

1 Figures in brackets indicate the literature references at the end of this paper

## References

[1] Fano U, Racah G (1959). Irreducible Tensorial Sets.

[2] Rotenberg M, Bivins R, Metropolis N, Wooten JK (1959). The 3—*j* and 6—*j* Symbols.

[3] Condon EU, Shortley GH (1951). The Theory of Atomic Spectra.

[4] Slater JC (1960). Quantum Theory of Atomic Structure.

